# IL-15 superagonist N-803 improves IFNγ production and killing of leukemia and ovarian cancer cells by CD34^+^ progenitor-derived NK cells

**DOI:** 10.1007/s00262-020-02749-8

**Published:** 2020-11-03

**Authors:** J. M. R. Van der Meer, R. J. A. Maas, K. Guldevall, K. Klarenaar, P. K. J. D. de Jonge, J. S. Hoogstad-van Evert, A. B. van der Waart, J. Cany, J. T. Safrit, J. H. Lee, E. Wagena, P. Friedl, B. Önfelt, L. F. Massuger, N. P. M. Schaap, J. H. Jansen, W. Hobo, H. Dolstra

**Affiliations:** 1 Department of Laboratory Medicine, Laboratory of Hematology, Radboud University Medical Center, Radboud Institute for Molecular Life Sciences, Geert Grooteplein Zuid 8, P.O. Box 9101, 6500 HB Nijmegen, The Netherlands; 2 Department of Applied Physics, Science for Life Laboratory, KTH - Royal Institute of Technology, Stockholm, Sweden; 3 NantKwest, Culver City, CA USA; 4 ImmunityBio, Culver City, CA USA; 5 Department of Cell Biology, Radboud Institute for Molecular Life Sciences, Nijmegen, The Netherlands; 6 David H. Koch Center for Applied Genitourinary Cancers, The University of Texas MD Anderson Cancer Center, Houston, TX USA; 7 Cancer Genomics Center, Utrecht, The Netherlands; 8 Department of Obstetrics and Gynecology, Radboud University Medical Center, Nijmegen, the Netherlands; 9 Department of Hematology, Radboud University Medical Center, Nijmegen, The Netherlands

**Keywords:** Natural killer cell, Ovarian cancer, Leukemia, N-803, IL-15 superagonist, Cancer immunotherapy

## Abstract

Allogeneic natural killer (NK) cell transfer is a potential immunotherapy to eliminate and control cancer. A promising source are CD34 + hematopoietic progenitor cells (HPCs), since large numbers of cytotoxic NK cells can be generated. Effective boosting of NK cell function can be achieved by interleukin (IL)-15. However, its in vivo half-life is short and potent trans-presentation by IL-15 receptor α (IL-15Rα) is absent. Therefore, ImmunityBio developed IL-15 superagonist N-803, which combines IL-15 with an activating mutation, an IL-15Rα sushi domain for trans-presentation, and IgG1-Fc for increased half-life. Here, we investigated whether and how N-803 improves HPC-NK cell functionality in leukemia and ovarian cancer (OC) models in vitro and in vivo in OC-bearing immunodeficient mice. We used flow cytometry-based assays, enzyme-linked immunosorbent assay, microscopy-based serial killing assays, and bioluminescence imaging, for in vitro and in vivo experiments. N-803 increased HPC-NK cell proliferation and interferon (IFN)γ production. On leukemia cells, co-culture with HPC-NK cells and N-803 increased ICAM-1 expression. Furthermore, N-803 improved HPC-NK cell-mediated (serial) leukemia killing. Treating OC spheroids with HPC-NK cells and N-803 increased IFNγ-induced CXCL10 secretion, and target killing after prolonged exposure. In immunodeficient mice bearing human OC, N-803 supported HPC-NK cell persistence in combination with total human immunoglobulins to prevent Fc-mediated HPC-NK cell depletion. Moreover, this combination treatment decreased tumor growth. In conclusion,  N-803 is a promising IL-15-based compound that boosts HPC-NK cell expansion and functionality in vitro and in vivo. Adding N-803 to HPC-NK cell therapy could improve cancer immunotherapy.

**Electronic supplementary material:**

The online version of this article (10.1007/s00262-020-02749-8) contains supplementary material, which is available to authorized users.

## Introduction

Natural killer (NK) cell therapy is an attractive strategy for cancer treatment as it selectively targets tumor cells without harming healthy tissues [1–3]. Moreover, numerous malignancies including hematopoietic and epithelial tumors are susceptible to NK cell-mediated immunity [4–8]. Since autologous NK cell infusion yields limited clinical responses [3], current approaches mostly involve allogeneic NK cell infusion in combination with cytokine support leading to improved responses [1, 2].

A promising source for allogeneic NK cell therapy are CD34^+^ hematopoietic progenitor cell (HPC)-derived NK cells, since large numbers of cytotoxic NK cells can be generated from various sources, including umbilical cord blood (UCB). First, CD34 + HPCs are expanded and subsequently differentiated into CD56^+^ HPC-NK cells, leading to more than 1000-fold expansion and high NK cell purity [9–11]. HPC-NK cells are highly functional, since they have high activating receptor expression, degranulation capacity, interferon (IFN)γ production, and tumor cell killing capacity [9–13]. Furthermore, we have shown that HPC-NK cells mediate anti-tumor responses in leukemia and ovarian cancer (OC) models in mice, leading to prolonged survival [11, 12, 14]. To further maximize the anti-tumor effects of HPC-NK cell therapy, combination treatments can be explored to maintain NK cell proliferation and activation and/or to augment NK cell-mediated killing of tumor cells.

Interleukin (IL)-2 is traditionally used to boost proliferation of adoptively transferred NK cells in vivo [1, 2, 15–17]. However, IL-2 has been shown to also expand regulatory T cells (Tregs) that may reduce NK cell functionality [18, 19]. Alternatively, IL-15 is crucial for NK cell survival, proliferation, and effector function [20, 21], but does not induce Treg expansion [22, 23]. Unfortunately, the in vivo half-life of recombinant IL-15 is short (≈40 min) [24]. Moreover, IL-15 is most potent when trans-presented by cells expressing the IL-15 receptor α (IL-15Rα) [25]. Hence, a novel IL-15 superagonist called N-803 (formerly known as ALT-803) has been developed, consisting of IL-15 with an activating mutation (N72D) that enhances binding to CD122 and CD122/CD132 activation, an IL-15Rα sushi domain to mimic typical trans-presentation, and an IgG1 Fc tail to increase half-life. N-803 has a more than 25-fold increased biological activity based on proliferation of 32Dβ cells [26] and more than 35-fold increased half-life (25 h) compared to IL-15 [24]. First reported clinical trials of N-803 in cancer patients revealed that it is well tolerated and stimulates NK cell activation and expansion [27, 28] and CD8^+^ T cells, but not Tregs [27]. In vitro, N-803 enhances functionality and tumor killing potential of peripheral blood (PB)-NK cells [29, 30] and ascites-derived NK cells [30, 31]. In vivo, PB-NK cell infusion in combination with N-803 administration results in significantly decreased tumor growth in NOD/SCID/IL2Rγnull (NSG) mice bearing human OC [30].

Our study goal was to investigate whether and how N-803 enhances HPC-NK cell functionality in leukemia and OC models, and whether N-803 supports HPC-NK cell persistence and anti-tumor effects in OC-bearing NSG mice. We found that N-803 can increase IFNγ production of HPC-NK cells and augment HPC-NK cell-mediated killing of OC and leukemia cells in vitro. Moreover, N-803 supports HPC-NK cell persistence and limits tumor growth in NSG mice bearing human OC.

## Materials and methods

### HPC-NK cell culture

UCB collection at delivery was approved (see “Compliance with ethical standards”). HPC-NK cells were generated as described [11] with the following minor modifications. Cells were cultured for 5–7 weeks in 6-well tissue culture plates (Corning, 3506), using NK MACS Basal medium and supplement (NK MACS, Miltenyi Biotec, 130–114-429) complemented with 10% human serum (HS, Sanquin) during expansion (day 0–14) and 2–10% HS during differentiation (from day 14). HPC-NK cells (> 70% CD56^+^) were used directly or cryopreserved. Cryopreserved HPC-NK cells were thawed and used after 5–7 days of culture in NK MACS containing 10% HS, 50 ng/ml recombinant human (rh)IL-15 (Immunotools, 11340155) and 0.2 ng/ml rhIL-12 (Miltenyi Biotec, 130–096-704). For experiments, HPC-NK cells were resuspended in Iscove's Modified Dulbecco's Medium (IMDM, Gibco, 21980–032) supplemented with 10% fetal calf serum (FCS, Integro, 5–45900 or Corning, 35–079-CV) (IMDM10), except assays with primary AML samples (10% HS), proliferation assays, and some serial killing experiments in microwells (NK MACS medium + 10% HS or FCS, respectively).

### PB-NK cell isolation

To obtain PB-NK cells, peripheral blood mononuclear cells were isolated from healthy donor buffy coats (Sanquin Blood Supply Foundation) by density gradient Ficoll-paque™ PLUS (Sigma-Aldrich, 17–1440-03) centrifugation. Next, PB-NK cells were isolated using a magnetic bead-based NK cell enrichment kit (StemCell Technologies, 19055) resulting in ≥ 90% purity. PB-NK cells were resuspended in IMDM10 for experiments.

### Tumor cell culture

OC cell lines SKOV-3, IGROV-1 and OVCAR-3 (RRID:CVCL_0532, RRID:CVCL_1304 and RRID:CVCL_0465, respectively) were cultured in Roswell Park Memorial Institute 1640 medium (RPMI, Gibco, 21875–034) supplemented with 10% FCS for SKOV-3 and IGROV-1 or 20% FCS and 1 µg/ml insulin (Merck, i0516) for OVCAR-3. SKOV-3 was transduced with luciferase (luc) and green fluorescent protein (GFP) (SKOV-3-luc-GFP) and cloned as described [11]*,* and used for killing assays. Leukemia cell lines K562 and THP-1 (RRID:CVCL_0004 and RRID:CVCL_0006, respectively) were cultured in IMDM10. All cell lines were cultured for maximally three months and were mycoplasma free. SKOV-3, K562, and THP-1 were purchased from ATCC, IGROV-1 and OVCAR-3 were provided by Prof. Dr. OC Boerman, Department of Nuclear Medicine, Radboudumc, Nijmegen, the Netherlands.

### Tumor spheroid generation

Spheroids were generated from SKOV-3 and SKOV-3-luc-GFP as described in Hoogstad-van Evert et al. [11] with the following adaptations. Culture medium was not supplemented with bovine serum albumin but with 10% FCS and 1% penicillin/streptomycin (MP Biomedicals, 1670049) and agarose medium with 2% penicillin/streptomycin. Tumor spheroids were used 3–5 days after initial seeding.

### Flow cytometry (FCM)-based assays

FCM samples were measured on one of the following flow cytometers: FC500, Gallios, CytoFLEX (all Beckman Coulter).

### NK cell proliferation

NK cells were labeled with eFluor450 (eBioScience, 65-0842-85) and cultured in NK MACS/10% HS with/without rhIL-15 or N-803 (ImmunityBio). Cytokines were refreshed on day 3 and FCM analysis was performed on day 3 and 6. Dead cells were excluded using Fixable Viability Dye eFluor780 (eBiosciences, 65-0865-18). The proliferation gate was set on 1% in the no cytokine condition on day 3. NK cell numbers were based on CD56 gating (CD56-PE-Cy7, Beckman Coulter, A21692) and measuring for a fixed time.

### Intercellular adhesion molecule 1 (ICAM-1) expression

Tumor cell lines and NK cells were plated at an effector-to-target (E:T) ratio of 0.6:1, with 0 or 1 nM N-803. After overnight-24 h co-incubation, cells were stained with antibodies CD56-PE-Cy7 (BioLegend, 318318), ICAM-1-FITC (Biolegend, 353108) (and CD15-PE (IQ Products, IQP-129R) for THP-1). Primary AML samples were labelled with 0.25 µM carboxyfluorescein diacetate succinimidyl ester (CFSE, Invitrogen, C1157), co-cultured with NK cells (E:T ratio 0.1:1 or 0.3:1) for 48 h and stained with CD33-BV605 (BD Biosciences, 740400) and ICAM-1 PE-Vio770 (Miltenyi Biotec, 130–104-031). Primary AML samples contained > 90% blasts based on CD33 expression. Obtaining primary AML cells and patient data at diagnosis was approved (see “Compliance with ethical standards”).

### IFNγ and perforin content

For IFNγ content, HPC-NK cells were stimulated for 4 h with K562, THP-1 or SKOV-3 at an E:T ratio of 1.5:1, in the absence or presence of 1 nM N-803, 1 nM rhIL-15, or 1000 U/ml rhIL-2 (Chiron, NDC 53905–991-01) and in the presence of brefeldin A (added after 1 h, BD Biosciences, 555029). For perforin production, PB-NK cells and HPC-NK cells were primed overnight with or without 1 nM N-803.

After stimulation, surface staining was performed of CD56-BV510 (Biolegend, 318340), and intracellular staining of perforin-PE (Biolegend, 308106) and IFNγ-FITC (BD Biosciences, 554700). Dead cells were excluded using Fixable Viability Dye eFluor780. IFNγ analysis was performed by gating on CD56^+^ perforin^+^ NK cells, using unstimulated cells as control. Perforin analysis was performed by gating on CD56^+^ NK cells.

### Killing assay

Targets were plated at 30,000 cells/well in 96-well plates (round-bottom for leukemia cells, flat-bottom for OC cells). Targets or HPC-NK/PB-NK cells were labeled with 0.25–1 µM CFSE, and co-cultured at different E:T ratios with or without 1 nM N-803. Notably, SKOV-3-luc-GFP was not labeled with CFSE. OC cells were plated 3 h in advance to allow for adherence. After overnight (cell line) or 48 h (primary cells) co-culture, supernatants were harvested and stored at − 20 °C for enzyme-linked immunosorbent assay (ELISA). Next, leukemia cells and/or NK cells were collected. OC cells were trypsinized using trypLE (Gibco, 12605028) and collected. Subsequently, viability marker 7-Aminoactinomycin D (7-AAD, Sigma, A9400) was added and targets were analyzed. Percentage of target killing by NK cells was calculated as follows: [1–(number of viable targets after co-culture with NK cells)/(number of viable targets cultured without NK cells) × 100%].

### Spheroid killing assay

For spheroid killing assays, SKOV-3-luc-GFP cells were used. For overnight assays, different HPC-NK cell numbers were added with or without 1 nM N-803. After co-culture, supernatant was collected for ELISA. For 7-day assays, 13,000 HPC-NK cells and 0, 0.1 or 1.0 nM N-803 or rhIL-15 was used and after 7 days HPC-NK cells were counted based on CD56 positivity and 7-AAD negativity. Spheroids were washed, disrupted using trypLE and targets were counted based on GFP positivity and 7-AAD negativity.

### Infiltration assay

SKOV-3-luc-GFP or SKOV-3 spheroids were co-cultured with 200,000 HPC-NK cells with or without 1 nM N-803. In SKOV-3 experiments, HPC-NK cells were labeled with 1 µM CFSE before or CD56-PE-Cy7 after co-culture. After 3 h co-culture, infiltrated and non-infiltrated NK cells were separated as described [32]. First, supernatant was collected containing non-infiltrated NK cells. Next, spheroids were washed, disrupted using trypLE, and infiltrated NK cells were collected. 7-AAD negative and CD56 or CFSE positive NK cells were counted.

### ELISA

Supernatants were thawed to evaluate IFNγ, granzyme B and C-X-C motif chemokine 10 (CXCL10) secretion by ELISA according to manufacturer’s instructions (IFNγ, Endogen, M700A; granzyme B, MABTECH, 3485-IH-6; and CXCL10, R&D Systems, DY266-05).

### NK cell serial killing experiments in microwells

Experiments were executed with small adaptations from Guldevall et al*.* [33]. HPC-NK cells were stained with 1 µM CFSE (BD Biosciences, 565082) or 2.5 µM CellTrace Yellow (Invitrogen, C34567). Targets were labeled with 1–2 µM Far Red (Invitrogen, C34564) and dead cells were detected by 1 µM sytox blue (Invitrogen, S11348) or 50 nM sytox green (Invitrogen, S7020). After a pre-screening with targets only in microwells (50 × 50 × 300 µm^3^), HPC-NK cells were stochastically seeded with or without 1 nM N-803, 1 nM rhIL-15 or 1000 U/ml rhIL-2. Screening lasted for 12 h, using an inverted confocal microscope equipped with × 10 objective (Zeiss, LSM 880) at 37 °C, 5% CO_2_, with an image captured every 6 h. Wells with or without N-803 were imaged in parallel by separating compartments of the chip using a polydimethylsiloxane gasket. Image analysis was performed with a MatLab script developed in-house. E:T ratios of 1:5 to 1:10 were analyzed. Only wells with 1 NK cell were analyzed.

### Organotypic 3D collagen matrix assay

Organotypic 3D collagen matrix assays were performed as described [34]. In brief, 7500 SKOV-3-luc-GFP cells were plated on a flat-bottom 96-well imaging plate (Greiner CELLSTAR^®^, 655090). After overnight adherence, 7500 HPC-NK cells were added in a collagen solution (75 µl/well PureCol1, Advanced Biomatrix, 5005, 3 mg/ml) containing no or 1 nM N-803. After polymerization, no or 1 nM N-803 was added and cells were imaged by time-lapse bright field microscopy with × 20 objective (BD, Pathway 855) at 37° C, 5% CO_2_. Images were captured every 70s for ~ 24 h and subsequently, manual analysis of single cells was performed. Only serial killers were analyzed, defined as NK cells killing two or more targets. Inclusion criteria for cytotoxic events were (i) contact occurred between a single NK cell to a single target, (ii) the target was visible from the start of the movie.

### Mouse experiments

Animal experiments were performed according to approved protocols (see “Compliance with ethical standards”). For experiment 1, 24 female NSG mice (Jackson Laboratories) of 6–20 weeks old were injected intraperitoneally (i.p.) with 0.2 million SKOV-3-luc-GFP cells (day − 4) and divided into four treatment groups based on block randomization after bioluminescence imaging (BLI) 3 days later (day − 1). On day 0, mice were infused i.p. with HPC-NK cells (12 million/mouse). From day 0–15, mice (average weight was 25 g) received i.p. injections of 50 or 200 µg/kg N-803 twice weekly, or 2.5 µg rhIL-15 (~7 × more molecules compared to 50 µg/kg N-803) or phosphate buffered saline (PBS) every 2 days. Mice were sacrificed at day 15 or 16. Then, a peritoneal wash was performed and NK cells were labeled with mCD45-AF700 (Biolegend, 103128), hCD45-KO (Beckman Coulter, B36294) and hCD56-PE-Cy7 (Biolegend, 318318) and counted by flow cytometry.

Experiment 2 had a similar design with the following adaptations: 30 NSG mice were divided into five treatment groups and on day − 5, two groups were irradiated with 2.25 Gy. From day -1 onwards, one group received i.p. nanogam (total human immunoglobulins, Sanquin Bloodbank) injections (50 mg) weekly 1 day before N-803 injection. From days 0–15, mice received i.p. injections of 50 µg/kg N-803 twice weekly or 2.5 µg rhIL-15 every 2 days. Prior to HPC-NK cell injection, CD16 expression was determined using viability dye eFluor780, CD56-BV510 (Biolegend, 318340) and CD16-BV421 (Biolegend, 302038). Mice were sacrificed at day 14 or 15.

Experiment 3 had a comparable design with the following differences: from day -1 onwards, all (21, divided into three treatment groups) mice received nanogam. On day 0 and 4, mice were infused with i.p. HPC-NK cells (8–9 million/mouse/infusion) or PBS. Mice receiving HPC-NK cells also received i.p. injections of 2.5 µg rhIL-15 every 2 days or 50 µg/kg N-803 twice weekly from day 0–24. BLI was performed weekly until signal saturation, following i.p. injection with 150 mg/kg D-luciferin (PerkinElmer, 122799) and isoflurane anesthesia. Ten minutes after injection, BLI images were collected in an In Vivo Imaging System using Living Image software. A region of interest was drawn around the torsos of the mice, and the integrated flux of photons (photons/second/cm^2^/steradian) was analyzed.

### Statistical analysis

Statistical analysis was performed using Graphpad Prism software version 5.03. Fold changes, lag phase to apoptosis and NK cell numbers in mice were first log transformed. Two-sided Student *t* tests and one-way and two-way ANOVAs were used as indicated in the figure legends. Significance was defined as *p* < 0.05 (*), *p* < 0.01 (**) and *p* < 0.001 (***).

## Results

### N-803 enhances HPC-NK cell proliferation, IFNγ production, and leukemia killing

**Fig. 1:**
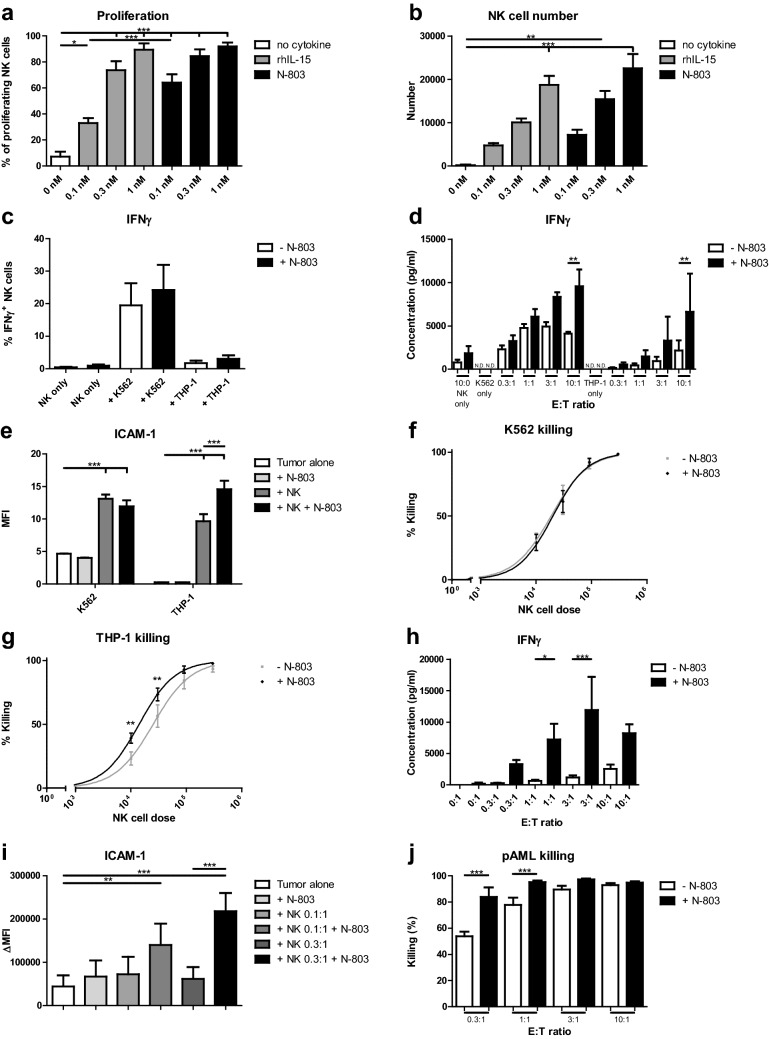
N-803 enhances HPC-NK cell proliferation, IFNγ production, and leukemia killing. **a–b** (**a**) Percentage of proliferating HPC-NK cells based on proliferation dye eFluor450 staining (*n* = 3–4), (**b**) number of HPC-NK cells based on CD56 antibody staining (*n* = 4–5) 6 days after incubation with no cytokine (white), rhIL-15 (grey) or N-803 (black). **c** Percentage of IFNγ^+^ HPC-NK cells 4 h after incubation with K562 or THP-1, with (black) or without (white) 1 nM N-803 combined for NK only (*n* = 6), K562 (*n* = 6) or THP-1 (*n* = 4). **d** IFNγ concentration (pg/ml) after overnight co-culture of HPC-NK cells and K562 (*n* = 3) or THP-1 (*n* = 4) with or without 1 nM N-803 (without, white; with N-803, black, ND = not detectable). **e** Geometric mean fluorescence intensity (MFI) of ICAM-1 expression after overnight culture of K562 (*n* = 3) or THP-1 (*n* = 4, white), and addition of N-803 (light grey), HPC-NK cells (dark grey) or both (black). **f**–**g** Percentage of (**f**) K562 (*n* = 3) or (**g**) THP-1 (*n* = 4) killing after overnight co-culture with HPC-NK cells and 0 (grey) or 1 nM N-803 (black). **h** IFNγ concentration (pg/ml) after 48 h co-culture of HPC-NK cells and primary AML (pAML) cells (*n* = 5) with 1 nM N-803 (black) or without cytokine (white). **i** Delta median fluorescence intensity (ΔMFI) of ICAM-1 expression after 48 h culture of pAML cells (*n* = 4, white), addition of N-803 (lightest grey), HPC-NK cells or both (different shades of grey/black) compared to a backbone sample for each condition. **j** Percentage of pAML cell killing (*n* = 5) after 48 h co-culture with HPC-NK cells and 0 (white) or 1 nM N-803 (black). Graphs show mean ± SEM for **a–g**, **i**–**j.** One-way ANOVA with Bonferroni correction was used (repeated measures for **f**–**g**, **i**–**j**) to test for statistical significance

Previously, we showed that N-803 outperforms rhIL-15 in inducing HPC-NK cell proliferation at 0.1 nM [35]. To confirm the optimal N-803 concentration, we performed proliferation assays with different concentrations of rhIL-15 or N-803 for 6 days. Indeed, N-803 induced HPC-NK cell proliferation in a dose-dependent manner (Fig. 1a, b). In comparison with rhIL-15, N-803 was superior in boosting NK cell proliferation at 0.1 nM (33–64%) and proliferation was similar at 1.0 nM (90–92%). All further experiments were performed using 1.0 nM N-803, which induced the most proliferation.

Next, we stimulated HPC-NK cells with leukemia cell lines K562 or THP-1 for 4 h with or without N-803 and analyzed IFNγ production. N-803 increased IFNγ production in the presence of K562 or THP-1 (Fig. 1c). RhIL-15 and rhIL-2 showed comparable effects as N-803 (Supplementary Fig. 1a). To investigate whether IFNγ secretion was augmented, we co-cultured HPC-NK cells with K562 or THP-1 overnight with/without N-803, harvested supernatants and performed ELISA. Accordingly, HPC-NK cell-mediated IFNγ secretion was enhanced by N-803 (Fig. 1d).

Since IFNγ promotes ICAM-1 expression on leukemia cells [36], HPC-NK cells were co-cultured overnight with K562 or THP-1 with or without N-803, whereupon ICAM-1 expression was analyzed. HPC-NK cell co-culture significantly upregulated ICAM-1 on K562 and THP-1, while N-803 treatment did not (Fig. 1e). Importantly, N-803 combined with HPC-NK cells further boosted ICAM-1 expression on THP-1.

As increased ICAM-1 expression stimulates NK cell-mediated killing due to strengthened interactions of NK cells and targets [36], we next investigated NK cell-mediated tumor killing. Leukemia killing was measured after overnight co-culture with HPC-NK cells and with or without N-803. Correlating with ICAM-1 expression, N-803 did not increase HPC-NK cell-mediated K562 killing, but significantly augmented HPC-NK cell-mediated THP-1 killing (Fig. 1f, g). To compare the killing capacity of HPC-NK cells and PB-NK cells, we co-cultured HPC-NK cells or PB-NK cells with K562 or THP-1 with or without N-803. For MHC-I negative K562, N-803 did not improve HPC-NK cell-mediated killing at all, while it did seem to improve PB-NK cell mediated killing at the second highest NK cell dose (Supplementary Fig. 1b). With regard to MHC-I positive THP-1, N-803 increased HPC-NK and PB-NK cell-mediated killing at all NK cell doses (Supplementary Fig. 1c). For both K562 and THP-1, HPC-NK cells were better killers than PB-NK cells at all NK cell doses, except the highest NK cell dose for K562 at which killing was maximal for both NK cell sources. Next, we evaluated the perforin content and granzyme B release of HPC-NK cells and PB-NK cells after priming with N-803 by intracellular staining and ELISA, respectively. We found that both HPC-NK cells and PB-NK cells upregulate perforin and granzyme B levels upon N-803 priming (Supplementary Fig. 1d, e). The higher killing capacity of HPC-NK cells did not correspond to perforin content, but did correlate with a higher granzyme B release versus PB-NK cells.

To confirm our findings, we co-cultured HPC-NK cells with primary AML samples from patients (Table 1) for 48 h with/without N-803 and investigated IFNγ production, ICAM-1 expression, and killing.

**Table 1:** Primary AML patient sample characteristics

AML#	Origin	FAB classification	% blasts
1	Bone marrow	M0	93
2	Bone marrow	M2	91
3	Bone marrow	M2	98
4	Bone marrow	M1	99
5	Bone marrow	M2	98

*FAB* French-American-British

N-803 significantly enhanced IFNγ production at an E:T ratio of 1:1 and 3:1 (Fig. 1h), upregulated ICAM-1 expression in the presence of HPC-NK cells (Fig. 1i) and most importantly increased primary AML killing by HPC-NK cells (Fig. 1j). Collectively, these data show that N-803 boosts IFNγ production by HPC-NK cells, promotes ICAM-1 expression on leukemia cells and improves HPC-NK cell-mediated leukemia killing.

### N-803 enhances serial killing properties of HPC-NK cells against leukemia

**Fig. 2:**
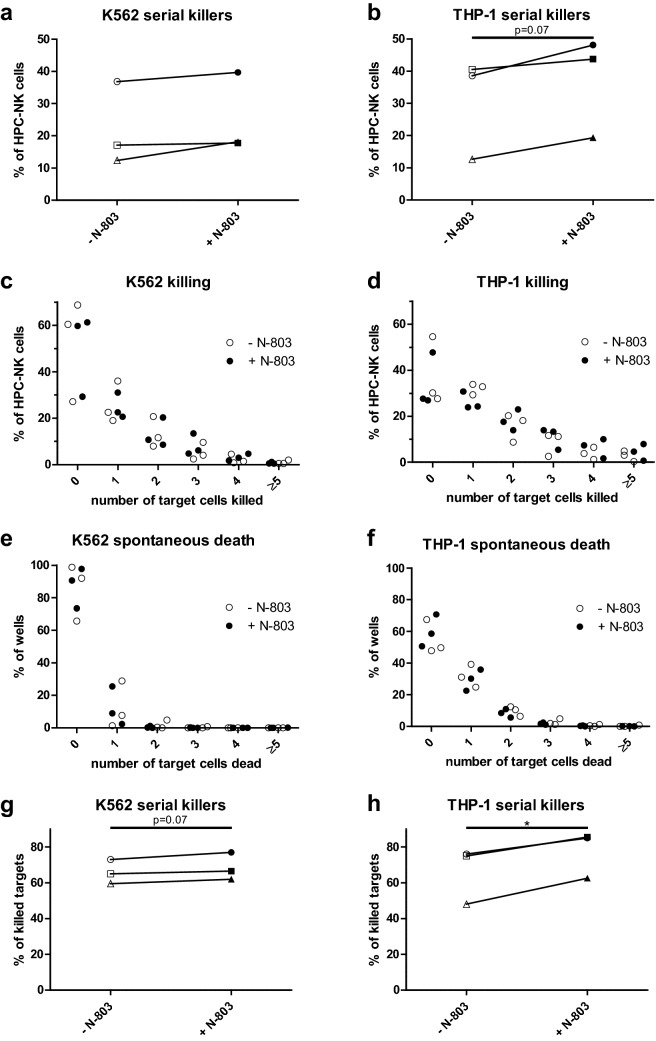
N-803 enhances serial killing properties of HPC-NK cells against leukemia. **a**–**b** Percentage of serial killers after 12 h co-culture of HPC-NK cells and (**a**) K562 (*n* = 3) or (**b**) THP-1 (*n* = 3) with 0 or 1 nM N-803. At least 125 NK cells were analyzed. **c–d** Percentage of wells showing the number of (**c**) K562 (*n* = 3) or (**d**) THP-1 (*n* = 3) cells killed in the presence of individual HPC-NK cells after 12 h co-culture with 0 (white) or 1 nM N-803 (black). At least 125 NK cells were analyzed. **e**–**f** Percentage of wells showing spontaneous (**e**) K562 (*n* = 3) or (**f**) THP-1 (*n* = 3) cell death after 12 h culture without HPC-NK cells and with 0 (white) or 1 nM N-803 (black). At least 173 targets were analyzed. **g**–**h** Percentage of killed (**g**) K562 (*n* = 3) or (**h**) THP-1 (*n* = 3) cells killed by serial killers after 12 h co-culture with HPC-NK cells and no cytokine or 1 nM N-803. At least 126 killed targets were analyzed. Graphs show mean ± SEM. Paired *t* tests were used for **a**-**b**, **g**–**h** and repeated measures one-way ANOVA with Bonferroni correction was used for **c**–**f** to test for statistical significance

To examine whether N-803 improves serial killing properties of HPC-NK cells against leukemia, we performed 12 h experiments using microwells for live cell imaging with single cell resolution [33]. Here, a mean of 22 or 31% of HPC-NK cells serially killed (≥ 2 targets with at least five targets present at *t* = 0 h) K562 and THP-1, respectively (Fig. 2a, b). N-803 seemed to enhance these percentages, most distinct for THP-1 (mean 37%, *p* = 0.07). Most killing HPC-NK cells killed 1 target, followed by 2, 3, 4 and 5 or more targets (Fig. 2c, d). N-803 seemed to increase the number of targets killed by HPC-NK cell serial killers, most pronounced for THP-1. Spontaneous target death was detected in the minority of wells without HPC-NK cells (mean 15% for K562, 45% for THP-1, Fig. 2e, f) and was not affected by N-803. Notably, the majority of targets was killed by serial killer HPC-NK cells (mean 66%, Fig. 2g, h). N-803 augmented this percentage to a mean of 69% for K562 and 78% for THP-1. RhIL-2 and rhIL-15 displayed similar results as N-803 (Supplementary Fig. 2). Together, these data demonstrate that N-803 improves serial killing properties of HPC-NK cells against leukemia.

### N-803 does not promote short-term HPC-NK cell-mediated killing of OC cell monolayers

**Fig. 3:**
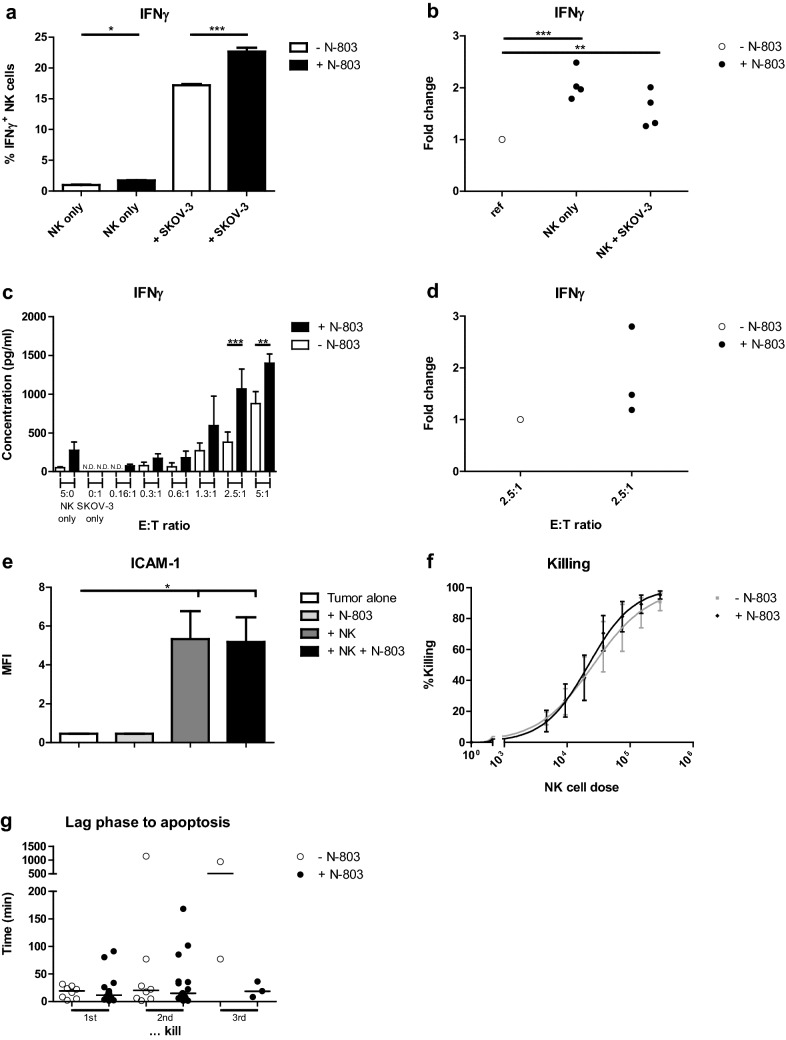
N-803 does not promote short-term HPC-NK cell-mediated killing of OC cell monolayers. **a**–**b** (**a**) Percentage of IFNγ^+^ HPC-NK cells or (**b**) fold change of the percentage after 4 h incubation with OC cell line SKOV-3, and 0 (white) or 1 nM N-803 (black) (**a**) in a representative HPC-NK cell donor containing duplos/triplos or (**b**) combined (*n* = 4) and compared to the same cells without cytokine (ref). **c**–**d** (**c**) IFNγ concentration (pg/ml) or (**d**) fold change of IFNγ concentration after overnight co-culture of HPC-NK cells and SKOV-3 with 0 (white) or 1 nM N-803 (black) (**c**) in a representative HPC-NK cell donor containing triplos (N.D. = not detectable) or (**d**) combined compared to without cytokine (*n* = 3). (**e**) MFI of ICAM-1 expression after overnight culture of SKOV-3 (white), addition of N-803 (light grey), HPC-NK cells (dark grey) or both (black) (*n* = 3). (**f**) Percentage of SKOV-3 killing after overnight co-culture with HPC-NK cells and 0 (grey) or 1 nM N-803 (black) (*n* = 4). (**g**) Lag phase to apoptosis of SKOV-3 for the 1st, 2nd and 3rd kill by serial killer HPC-NK cells with 0 (white) or 1 nM N-803 (black) (*n* = 1). Graphs show mean ± SD for **a**, **c**/SEM for **e**–**f**, and median for **g**. One-way ANOVA with Bonferroni correction was used (after log transformation for **b**, **d**, **g**, repeated measures for **b**, **d**–**f**) to test for statistical significance

To investigate whether N-803 also enhances the HPC-NK cell functionality towards OC cells, we stimulated HPC-NK cells with OC cell line SKOV-3 with/without N-803 for 4 h and evaluated IFNγ production. Similar to leukemia, IFNγ production significantly increased by N-803, for HPC-NK cells with and without SKOV-3 (Fig. 3a, b): median 1.5-fold for HPC-NK cells + SKOV-3, *p* < 0.01. Accordingly, IFNγ secretion determined by ELISA was slightly increased by N-803 (Fig. 3c-d). However, no ICAM-1 upregulation was observed on SKOV-3 after addition of HPC-NK cells and N-803, compared to addition of HPC-NK cells alone (Fig. 3e). Likewise, N-803 did not improve HPC-NK cell-mediated SKOV-3 killing (Fig. 3f). OC cell lines IGROV-1 and OVCAR-3 showed similar killing results as SKOV-3 (Supplementary Fig. 3); rhIL-15 displayed comparable overnight IFNγ production and SKOV-3 killing as N-803 (data not shown).

Although N-803 did not improve overnight HPC-NK cell-mediated SKOV-3 killing, we next studied whether interaction abilities and serial killing properties of HPC-NK cells against OC were affected by N-803 in an organotypic 3D collagen matrix assay, mimicking interstitial tissue. Lag phase to SKOV-3 apoptosis (time from first contact to kill) due to serial killing by HPC-NK cells was mostly short with a median of 19 min for the first kill and similar times for the second kill (Fig. 3g). N-803 did not change these times for the first or second kill. Altogether, these data indicate that despite slightly enhanced IFNγ production, N-803 could not increase ICAM-1 expression and short-term HPC-NK cell-mediated (serial) killing of OC cells.

### N-803 increases CXCL10 production and improves long-term HPC-NK cell-mediated killing in OC spheroids

**Fig. 4:**
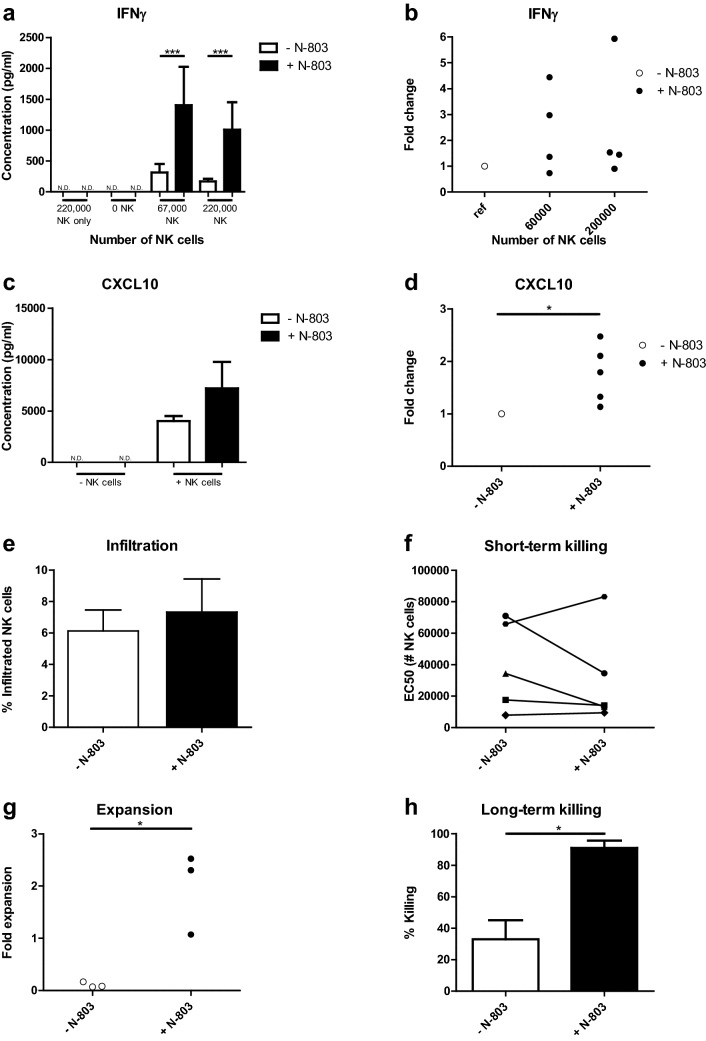
N-803 increases CXCL10 production and improves long-term HPC-NK cell-mediated killing in OC spheroids. **a–b** (**a**) IFNγ concentration (pg/ml) or (**b**) fold change of IFNγ concentration after overnight co-culture of HPC-NK cells and spheroids of OC cell line SKOV-3 with 0 (white) or 1 nM N-803 (black) (**a**) in a representative HPC-NK cell donor containing fiveplos/sixplos (N.D. = not detectable) or (**b**) combined (*n* = 4) and compared to the same number of NK cells without cytokine (ref, 55,600–67,000 and 170,000–220,000 HPC-NK cells). **c**–**d** (**c**) CXCL10 concentration (pg/ml) or (**d**) fold change of CXCL10 concentration after overnight co-culture of HPC-NK cells and SKOV-3 spheroids with 0 (white) or 1 nM N-803 (black) (**c**) in a representative HPC-NK cell donor containing triplos (0–67,000 HPC-NK cells, N.D. = not detectable) or (**d**) combined (*n* = 5) compared to no cytokine (6700–67,000 HPC-NK cells). (**e**) Percentage of infiltrated HPC-NK cells into SKOV-3 spheroids after 3 h co-incubation with 0 or 1 nM N-803 (*n* = 8). (**f**) EC50 = HPC-NK cell dose needed to kill 50% of SKOV-3 in a spheroid with or without N-803 (*n* = 5). **(g**) Fold expansion of HPC-NK cells in the presence of a SKOV-3 spheroid after 1 week co-incubation with 0 or 1 nM N-803 (*n* = 3). **h** Percentage of SKOV-3 spheroid killing after 1 week co-culture with HPC-NK cells and 0 or 1 nM N-803 (*n* = 4). Graphs show mean ± SD for **a**, **c**/SEM for **e**, **h**. *T* tests were used for **c–h** (paired for **e–h**, unpaired for **c**, one-sample for **d**, after log transformation for **d** and **g** and a repeated measures one-way ANOVA with Bonferroni correction for **a** and **b** (after log transformation for **b** to test for statistical significance

Next, we addressed the effects of N-803 in SKOV-3(-luc-GFP) spheroids to mimic three-dimensional growth of OC in vivo. HPC-NK cells were co-cultured with spheroids overnight and ELISA of supernatants was performed to determine IFNγ and CXCL10 secretion. Overall, N-803 enhanced IFNγ secretion of HPC-NK cells co-cultured with spheroids (Fig. 4a, b). Furthermore, CXCL10 production was significantly boosted by spheroids co-cultured with HPC-NK cells and N-803 (Fig. 4c, d). Since CXCL10 attracts C-X-C chemokine receptor 3 (CXCR3)^+^ HPC-NK cells [14], we performed 3 h infiltration assays with spheroids and HPC-NK cells, in which no effect of N-803 on infiltration was observed (Fig. 4e). Moreover, N-803 did not improve HPC-NK cell-mediated spheroid killing within 24 h (Fig. 4f). All short-term assays with rhIL-15 or rhIL-2 showed comparable results as N-803 (data not shown). Importantly, a long-term killing assay showed that N-803 significantly enhanced HPC-NK cell expansion and HPC-NK cell-mediated spheroid killing (Fig. 4g-h). A dose-dependent killing effect of N-803 was found and rhIL-15 displayed similar effects as N-803 (Supplementary Fig. 4). Collectively, these experiments demonstrate that N-803 increases IFNγ and CXCL10 secretion in co-cultures of OC spheroids and HPC-NK cells. Furthermore, N-803 induces HPC-NK cell expansion and boosts OC spheroid destruction during long-term co-cultures.

### HPC-NK cells combined with N-803 and nanogam show anti-tumor effects in mice bearing human OC

**Fig. 5:**
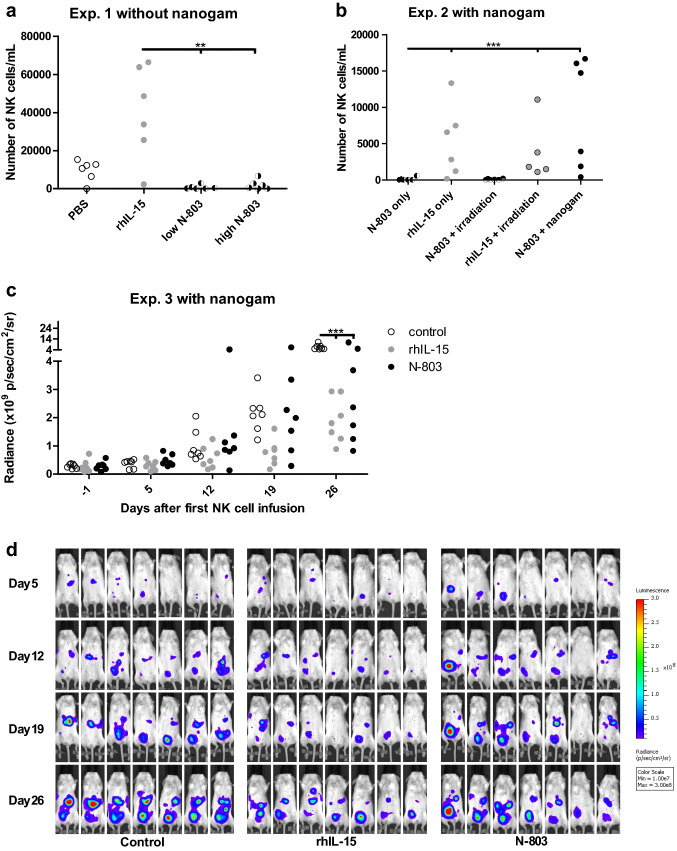
HPC-NK cells combined with N-803 and nanogam show anti-tumor effects in mice bearing human OC. (**a**) Experiment 1: number of HPC-NK cells per ml in the peritoneal wash of SKOV-3 bearing NSG mice (0.2 million tumor cells injected 4 days before HPC-NK cell treatment) 15/16 days after treatment with HPC-NK cells in combination with phosphate-buffered saline (PBS), 2.5 µg rhIL-15, or 50 or 200 µg/kg N-803 (6 mice per group). **(b**) Experiment 2: number of HPC-NK cells per ml in the peritoneal wash of SKOV-3 bearing NSG mice (0.2 million tumor cells injected 4 days before HPC-NK cell treatment) 14/15 days after treatment with HPC-NK cells in combination with 50 µg/kg N-803, 2.5 µg rhIL-15, 50 µg/kg N-803 + nanogam, 50 µg/kg N-803 + 2.25 Gy irradiation, or 2.5 µg rhIL-15 + 2.25 Gy irradiation (6 mice per group). **(c**) Experiment 3: radiance (photons/second/cm^2^/steradian) of tumors in SKOV-3 bearing NSG mice (0.2 million tumor cells injected 4 days before the first HPC-NK cell infusion) treated with nanogam + PBS (white, control), nanogam + 2 HPC-NK cell infusions + 2.5 µg rhIL-15 (grey) or nanogam + 2 HPC-NK cell infusions + 50 µg/kg N-803 (black) over time (7 mice per group). **(d**) BLI images from (**c**) acquired over time. One-way ANOVA with Bonferroni correction was used for **a**–**b** (after log transformation for **a** and **b**), and a repeated-measures two-way ANOVA with Bonferroni correction for **c** to test for statistical significance

To determine whether N-803 promotes HPC-NK cell persistence and anti-tumor effects in a human OC mouse model, we used NSG mice bearing peritoneal SKOV-3-luc-GFP tumor nodules [11]. In experiment 1, mice were treated i.p. with HPC-NK cells in combination with PBS, rhIL-15, or N-803 for two weeks and afterwards peritoneal washes were performed. As expected, HPC-NK cells were present in the rhIL-15 group but surprisingly HPC-NK cells were nearly absent in the N-803 groups (Fig. 5a). We hypothesized that the Fc part of N-803 binds to Fc receptors, resulting in Fc-mediated HPC-NK cell depletion, in NSG mice lacking immunoglobulins. Hence, in experiment 2 we used irradiation or nanogam (*i.e.,* total human immunoglobulins) to kill or inactivate immune cells containing Fc receptors present in NSG mice, or to block Fc receptors, respectively, to prevent Fc-mediated HPC-NK cell depletion in the presence of N-803. To determine if there was risk for Fc-mediated fratricide, CD16 expression was determined prior to HPC-NK cell injection, which showed 20% CD16^+^ HPC-NK cells (Supplementary Fig. 5). Irradiation could not prevent N-803-mediated depletion but nanogam could, resulting in HPC-NK cell persistence and similar NK cell numbers as rhIL-15 treatment (Fig. 5b).

Finally, we evaluated tumor growth (experiment 3) in mice treated with two i.p. HPC-NK cell injections in combination with N-803 or rhIL-15, and nanogam compared to a group only receiving nanogam. This experiment showed that both combination treatments significantly reduced tumor growth, compared to the control group (Fig. 5c, d). To conclude, we demonstrate that nanogam restores HPC-NK cell persistence in OC bearing NSG mice receiving N-803. Importantly, HPC-NK cell, N-803 and nanogam combination treatment has an anti-OC effect in vivo.

## Discussion

Allogeneic NK cell therapy is a promising approach for cancer treatment and HPC-NK cells mediate anti-tumor responses in leukemia and OC models [11, 12, 14]. However, tumor eradication is not complete in xenograft NSG models, indicating room for improvement. Optimizing HPC-NK cell anti-tumor efficacy can be achieved by cytokine co-administration. This study investigated whether and how IL-15 superagonist N-803 improves HPC-NK cell functionality in leukemia and OC models, and whether N-803 supports in vivo HPC-NK cell persistence and anti-OC effects.

First, we confirmed that N-803 dose dependently induces HPC-NK cell proliferation. Compared to rhIL-15, N-803 leads to higher proliferation at 0.1 nM but not 1.0 nM, caused by reaching maximum proliferation, which is in line with previous reports [30, 35]. Next, we demonstrated that N-803 improves IFNγ production of HPC-NK cells. This effect has been demonstrated in numerous NK cell studies [29, 30, 37–42]. In addition, N-803 increases ICAM-1 expression on leukemia cells after HPC-NK cell co-culture and improves (serial) leukemia killing. Since HPC-NK cells have high lymphocyte function associated antigen 1 (LFA-1) expression [11, 12, 35], the receptor for ICAM-1, the interaction strength between HPC-NK cells and targets is dependent on ICAM-1 expression. Increased ICAM-1 expression leads to stronger interactions, resulting in targets being more sensitive to killing [12, 36]. Interestingly, these effects were found with primary AML and THP-1, but not K562, which may be attributed to unaffected ICAM-1 expression on K562. Furthermore, K562 is MHC-I negative, making it very sensitive to NK cell-mediated killing. Since HPC-NK cells are highly potent killers compared to PB-NK cells, this leaves a narrow window for improvement. However, for less susceptible MHC-I positive THP-1 cells HPC-NK and PB-NK cell-mediated killing could be improved by N-803. Importantly, we showed for the first time that N-803 promotes HPC-NK cell serial killing properties and that some HPC-NK cells kill 5 or more leukemia cells within 12 h. This is in line with studies using PB-NK or NK-92 cells, in which up to 6 [43], 7 [44], 8 [33], or 14 [45] serial kills were reported within 6–16 h. Our findings further revealed that a minority of HPC-NK cells is a serial killer, responsible for the majority of killing. This is in accordance with previous studies [33, 44].

Moreover, we assessed HPC-NK cell serial killing properties against OC cells. As expected based on OC monolayer killing experiments, N-803 did not improve serial killing against OC. Nevertheless, serial killer HPC-NK cells generally kill quickly (median 19 min for the first kill) after initial contact. This median lag phase is similar as in Vanherberghen’s study [44], where the mean lag phase (time to lytic hit + time to death) was 17.5 min for serial killers. In our OC model, serial killer HPC-NK cells kill up to three targets, which is lower than our leukemia model and other studies [33, 43–45]. Potential explanations for those differences are that we used a low target density and a high E:T ratio in the OC model, while in our leukemia model and other studies higher target densities and/or lower E:T ratios were used. For low target cell densities, we and others [43, 45] observed that NK cells often stay in contact with apoptotic cells, limiting the number of serial kills. Lower E:T ratios allow for better serial killing detection, because every NK cell can kill more targets. Furthermore, intrinsic differences between used targets impact sensitivity to (serial) killing by NK cells [33, 45]. For instance, SKOV-3 used in our OC model is more difficult to kill than K562 used in our leukemia model and other studies (Figs. 1, 3).

In OC spheroids, N-803 significantly increases IFNγ and CXCL10 secretion during overnight co-culture with HPC-NK cells. Because HPC-NK cells have high CXCR3 expression [11–14, 35], increased CXCL10 secretion could improve NK cell infiltration. Since the relatively high amount of HPC-NK cells, needed for infiltration assays, destroys OC spheroids after overnight incubation, we measured infiltration after 3 h. In this model, no effect of N-803 on HPC-NK cell infiltration was observed, though 3 h co-incubation is likely too short to increase IFNγ and CXCL10 secretion and impact HPC NK cell infiltration. Importantly, in long-term assays, using less HPC-NK cells, N-803 improves HPC-NK cell expansion, and, therefore, OC spheroid killing at the longer term.

Finally, we showed that in vivo N-803 supports peritoneal HPC-NK cell persistence in the presence of human immunoglobulins (nanogam) in NSG mice bearing human OC and this combination treatment has an anti-OC effect. Similar findings were reported by Felices et al. [30], demonstrating improved OC tumor control in NSG mice treated with PB-NK cells in combination with N-803 compared to no treatment or NK cells alone. Since HPC-NK cells hardly persist without cytokine support, and clinical trials will be conducted with cytokine support, we chose to compare HPC-NK cells plus N-803 (or rhIL-15) treatment to no treatment. Notably, pre-treatment of NSG mice with human immunoglobulins (nanogam) was required to prevent Fc-mediated HPC-NK cell depletion by N-803 treatment. In patients, pre-treatment with nanogam will not be necessary, since they have immunoglobulins. In Felices’ study sublethal irradiation (2.25 Gy) was sufficient to prevent Fc-mediated depletion of PB-NK cells, while in our study sublethal irradiation (2.25 Gy) did not rescue Fc-mediated depletion of HPC-NK cells. One of the differences in the design of these two studies is the timing of irradiation: we irradiated the mice one day before tumor injection, while they irradiated the mice one day before NK cell injection. It might be that in our study immune cells containing Fc receptors in the NSG mice recovered or repopulated before the first N-803 injection, which could have led to Fc-mediated depletion. Alternatively, it could be that HPC-NK cells are more sensitive to Fc-mediated NK cell depletion than PB-NK cells due to differences in activation status. Around 20% of the HPC-NK cells had CD16 expression before NK cell injection (Supplementary Fig. 5), indicating that Fc-mediated fratricide might have been possible. Moreover, we know from our previous publications that CD16 expression is upregulated in NSG mice in vivo [12, 14], increasing the risk for Fc-mediated fratricide. Fortunately, Fc-mediated depletion of HPC-NK cells could be prevented by nanogam injection in NSG mice.

Comparing N-803 with rhIL-15 shows that in vivo OC growth was similar. However, it is important to note that the amount of molecules per dose was ~ 7 × lower for N-803 than rhIL-15 and rhIL-15 was given more frequently. Assuming all N-803 or rhIL-15 was consumed before the next dose administration, this suggests that N-803 may indeed have a higher biological activity compared to rhIL-15. In vivo experiments with leukemia-bearing NSG mice, NK cells, and N-803 have previously been carried out [35, 39]. Wagner et al. showed K562 leukemia control by N-803-primed PB-NK cells [39] and Cany et al. demonstrated intra-femoral THP-1 leukemia control by HPC-NK cells, N-803, and decitabine [35]. Since we found HPC-NK cell depletion in our i.p. OC model, repeating Cany’s leukemia study with nanogam might improve treatment results in mice.

Collectively, our results imply that N-803 is an attractive compound to promote HPC-NK cell expansion and functionality for NK cell therapy. Currently, two phase 1 clinical trials with N-803 are recruiting patients in the US in various cancer types (NCT03054909 and NCT02890758). In addition, N-803 has been shown to enhance antibody-dependent cellular cytotoxicity in vitro [38, 41] and checkpoint blockade therapy in cancer-bearing mice [40]. For future studies, it would be interesting to compare N-803 to the standard IL-2 co-administration with NK cell adoptive transfer for anti-tumor efficacy, to evaluate whether IL-2 can be replaced by N-803 to prevent Treg-expansion in cancer patients.

In conclusion, N-803 boosts HPC-NK cell proliferation and IFNγ production in vitro. Furthermore, N-803 improves (serial) leukemia killing and long-term OC spheroid destruction by HPC-NK cells. In vivo, N-803 in combination with human immunoglobulins supports HPC-NK cell persistence in NSG mice and this combination treatment mediates an anti-OC effect. In conclusion, N-803 is a promising IL-15-based compound to improve NK cell-based cancer immunotherapy.

### Electronic supplementary material

Below is the link to the electronic supplementary material.

Supplementary file1 (PDF 2179 kb)

## Data Availability

Not applicable.

## Abbreviations

NK: Natural killer
HPC: Hematopoietic progenitor cell
UCB: Umbilical cord blood
IFN: Interferon
OC: Ovarian cancer
AML: Acute myeloid leukemia
IL: Interleukin
Treg: Regulatory T cell
IL-15Rα: IL-15 receptor α
PB: Peripheral blood
NSG: NOD/SCID/IL2Rγnull
i.p.: Intraperitoneal(ly)
ICAM-1: Intercellular adhesion molecule 1
E:T Ratio: Effector-to-target ratio
LFA-1: Lymphocyte function-associated antigen 1
CXCL10: C-X-C motif chemokine 10
CXCR3: C-X-C chemokine receptor 3
MFI: Geometric mean fluorescence intensity
