# Supplementary material for: IL-15 superagonist N-803 improves IFNγ production and killing of leukemia and ovarian cancer cells by CD34+ progenitor-derived NK cells
Source: Cancer Immunol Immunother. 2020 Nov 3;70(5):1305–21. doi: 10.1007/s00262-020-02749-8 (PMC8053152; doi:10.1007/s00262-020-02749-8)
Supplement: Supplementary file 1 — Supplementary file1 (PDF 2179 kb) [file 262_2020_2749_MOESM1_ESM.pdf]

Supplementary Fig. 1

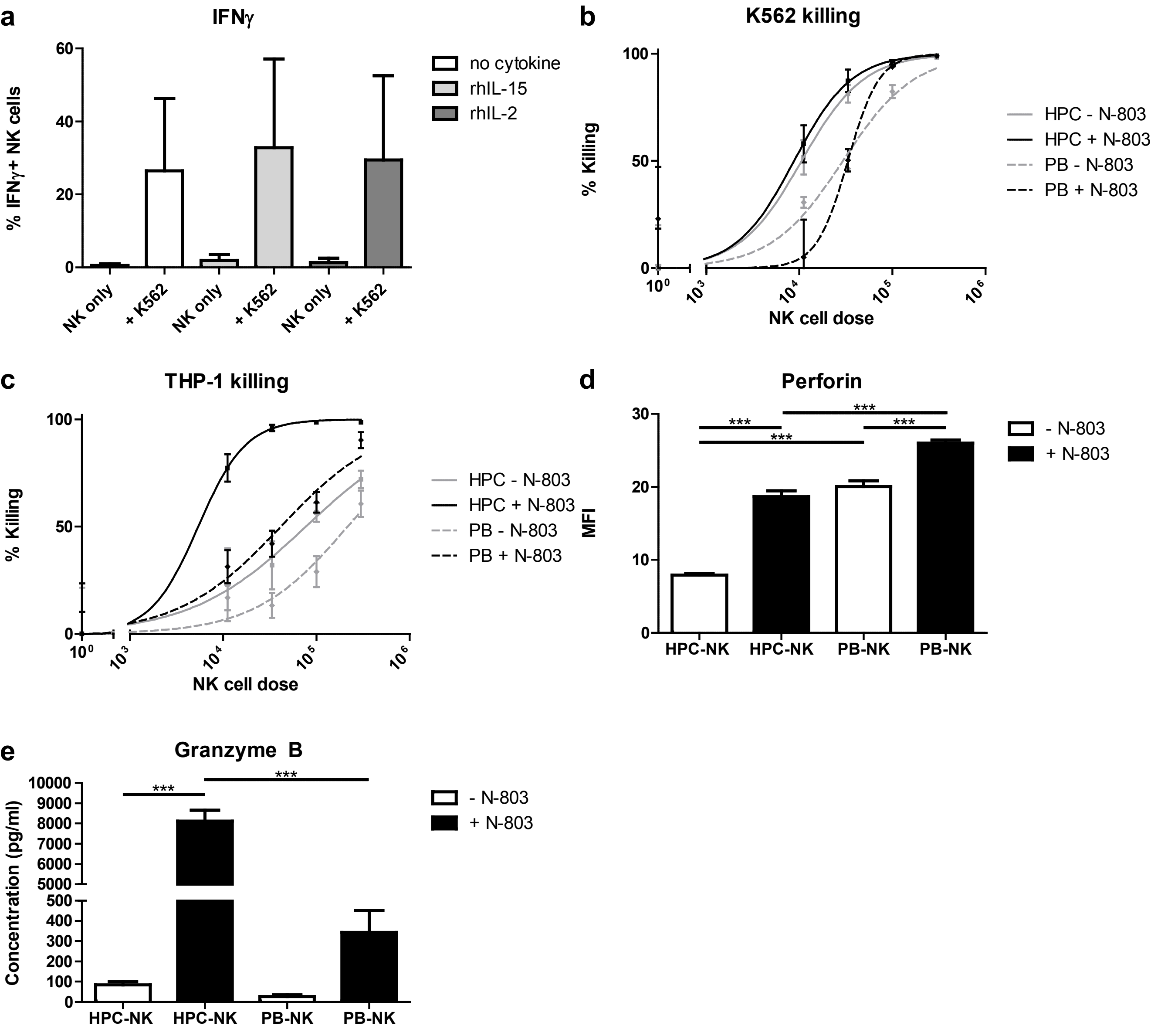

Supplementary Fig. 1: rhIL-15 and rhIL-2 increase IFNγ production in the presence of K562 and HPC-NK cells are better killers compared to PB-NK cells which correlates with a higher granzyme B release.

A. Percentage of IFNγ+ HPC-NK cells 4h after incubation with leukemia cell line K562 and no cytokine (white), 1 nM rhIL-15 (light grey) or 1000 U/ml rhIL-2 (dark grey) (n=2).

B-C. Percentage of (B) K562 or (C) THP-1 killing after overnight co-culture with HPC-NK cells (continuous line) or PB-NK cells (dashed line) and 0 (grey) or 1 nM N-803 (black) (n=1, triplos).

D. MFI of perforin expression in HPC-NK cells or PB-NK cells after overnight priming with or without 1 nM N-803.

E. Granzyme B concentration (pg/ml) after overnight priming of HPC-NK cells and PB-NK cells with or without 1 nM N-803.

Graph shows mean ± SEM for A/SD for B-E. One-way ANOVA with Bonferroni correction was used for A, D-E (repeated measures for A) to test for statistical significance.

Supplementary Fig. 2

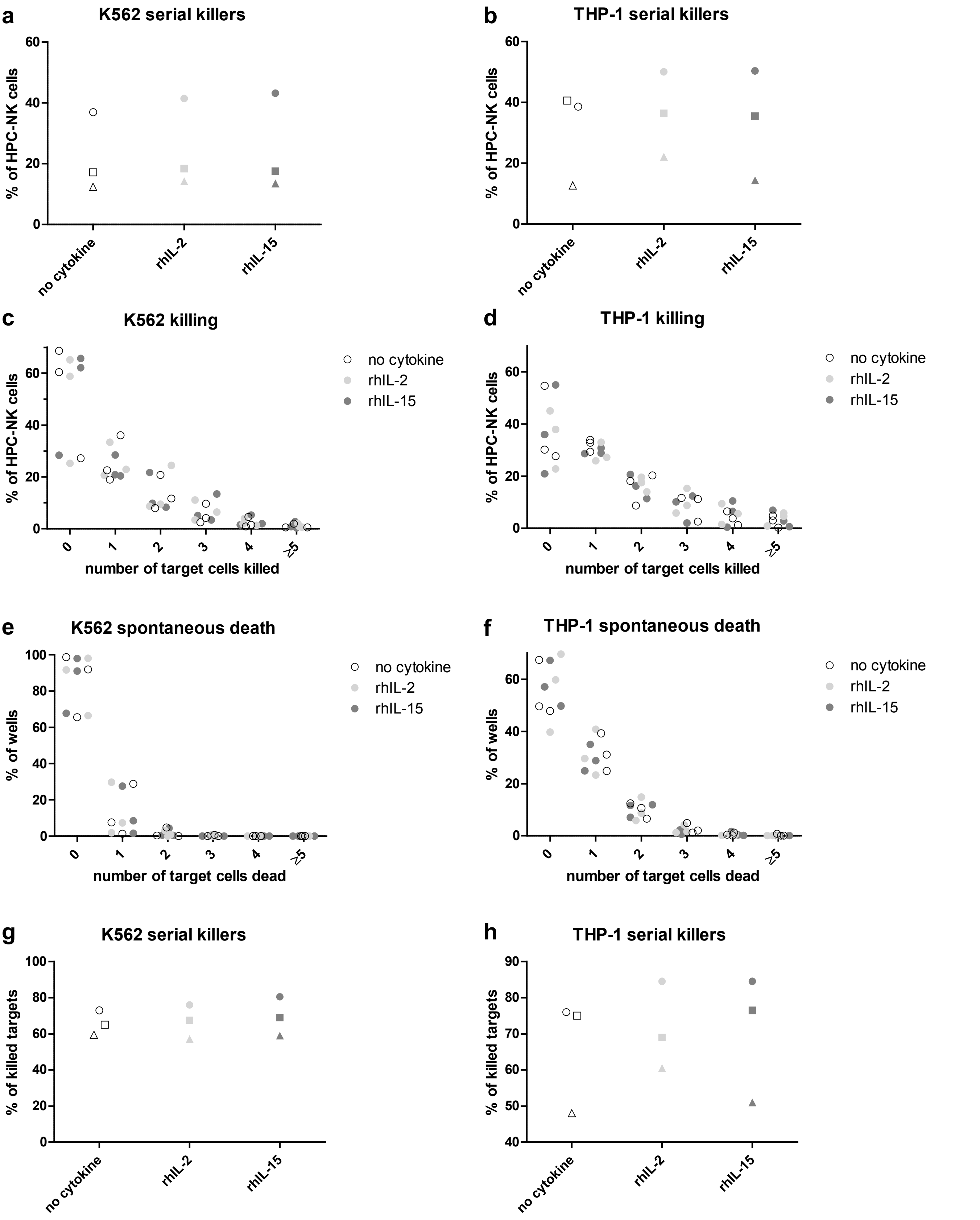

Supplementary Fig. 2: rhIL-2 and rhIL-15 show similar serial killing properties of HPC-NK cells against leukemia.
A-B. Percentage of serial killers after 12h co-culture of HPC-NK cells and (A) K562 (n=3) or (B) THP-1 (n=3) with no cytokine, 1000 U/ml rhIL-2, or 1 nM rhIL-15. At least 125 NK cells were analyzed.
C-D. Percentage of wells showing the number of (C) K562 (n=3) or (D) THP-1 (n=3) cells killed in the presence of individual HPC-NK cells after 12h co-culture with no cytokine (open), 1000 U/ml rhIL-2 (light grey), or 1 nM rhIL-15 (dark grey). At least 125 NK cells were analyzed.
E-F. Percentage of wells showing spontaneous (E) K562 (n=3) or (F) THP-1 (n=3) cell death after 12h culture without HPC-NK cells and with no cytokine (open), 1000 U/ml rhIL-2(light grey) or 1 nM rhIL-15 (dark grey). At least 173 target cells were analyzed.
G-H. Percentage of killed (G) K562 (n=3) or (H) THP-1 (n=3) cells killed by serial killers after 12h co-culture with HPC-NK cells and with no cytokine, 1000 U/ml rhIL-2, or 1 nM rhIL-15. At least 126 killed target cells were analyzed.
Graphs show mean ± SEM. Repeated measures one-way ANOVA with Bonferroni correction was used for A-B, G-H and repeated measures two-way ANOVA with Bonferroni correction for C-F to test for statistical significance.

Supplementary Fig. 3

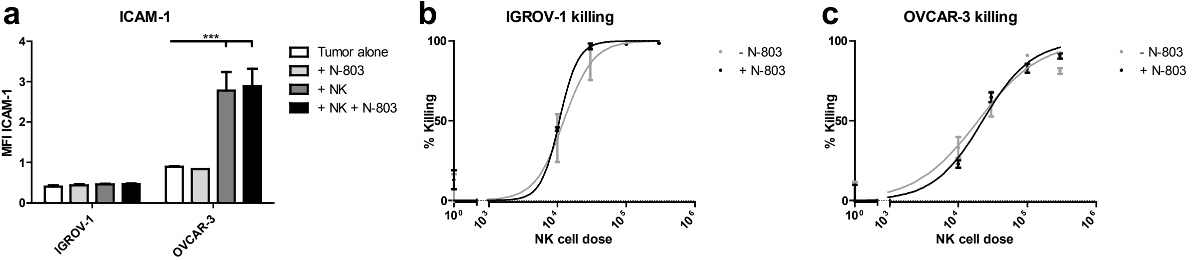

Supplementary Figure 3: N-803 does not increase HPC-NK cell-mediated killing of OC cells in monolayers overnight.
(A) MFI of ICAM-1 expression after overnight culture of OC cell lines IGROV-1 and OVCAR-3, addition of N-803 and/or HPC-NK cells (n=3). B-C. Percentage of (B) IGROV-1 or (C) OVCAR-3 killing after overnight co-culture with HPC-NK cells and no cytokine (grey) or 1 nM N-803 (black) (n=1 containing quadriplos). Graphs show mean ± SD for B-C/SEM for A. One-way ANOVA with Bonferroni correction was used for all subfigures (repeated measures for A) to test for statistical significance.

Supplementary Fig. 4

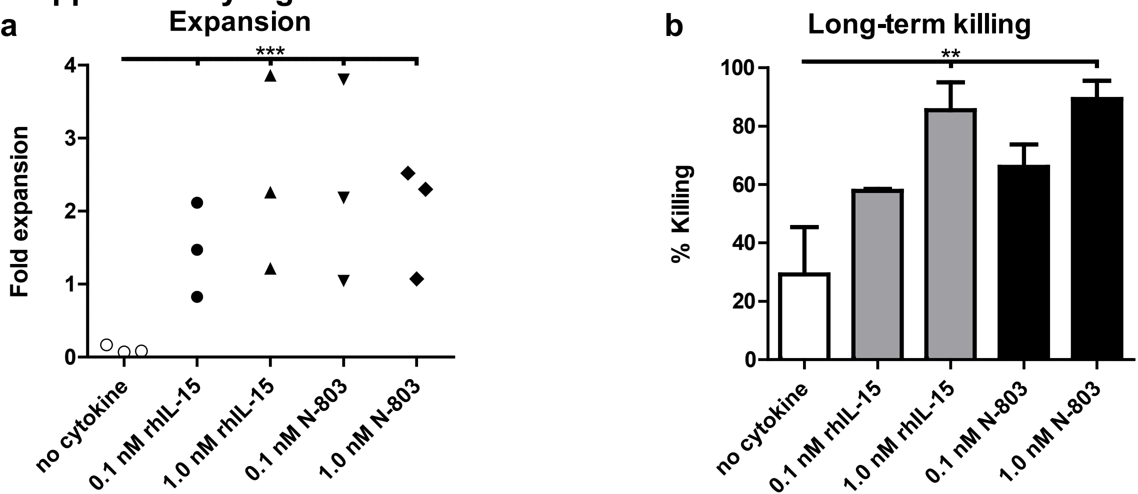

Supplementary Figure 4: rhIL-15 and N-803 expand HPC-NK cells and dose-dependently increase HPC-NK cell-mediated long-term OC spheroid killing.

A. Fold expansion of HPC-NK cells in the presence of a SKOV-3 spheroid after 1 week co-incubation with 0.1 or 1 nM rhIL-15 or N-803 or no cytokine (n=3).

B. Percentage of SKOV-3 spheroid killing after 1 week co-culture with HPC-NK cells and no cytokine, or 0.1 or 1 nM rhIL-15 or N-803 (n=3).

Graph B shows mean ± SEM. Repeated measures one-way ANOVA with Bonferroni correction was used (after log transformation for A) to test for statistical significance.

Supplementary Fig. 5

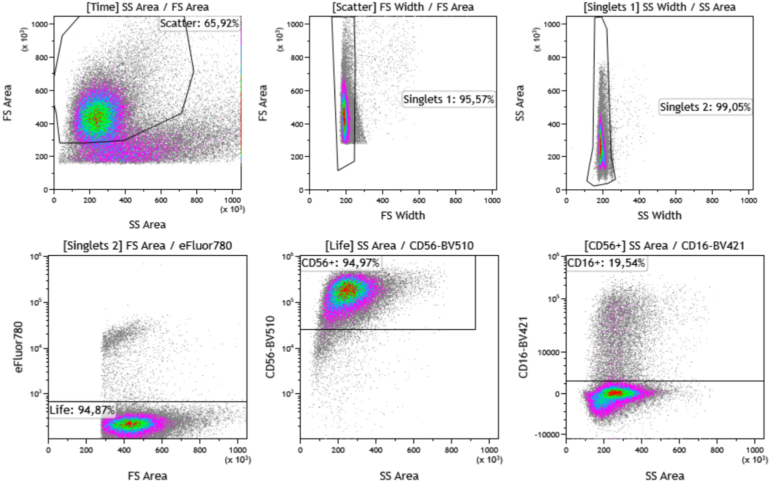

Supplementary Figure 5: CD16 expression in HPC-NK cells before injection into NSG mice. The gating strategy was based on time, lymphocyte size, doublet discrimination and dead cell exclusion using viability dye eFluor780. Next, $CD56^{+}$ cells were gated and CD16 expression was determined.
